# The effect of perceptual organization on numerical and preference-based decisions shows inter-subject correlation

**DOI:** 10.3758/s13423-022-02234-6

**Published:** 2023-01-10

**Authors:** Moshe Glickman, Tal Sela, Marius Usher, Dino J. Levy

**Affiliations:** 1 Department of Experimental Psychology, University College London, London, UK; 2 Max Planck UCL Centre for Computational Psychiatry and Ageing Research, University College London, London, UK; 3 Department of Behavioral Sciences, Kinneret Academic College, Tzemach, Israel; 4 The School of Psychological Sciences, Tel Aviv University, Tel Aviv, Israel; 5 Sagol School of Neuroscience, Tel Aviv University, Tel Aviv, Israel; 6 Coller School of Management, Tel Aviv University, Tel Aviv, Israel

**Keywords:** Risky choice, Numerical cognition, Computational modeling, Perceptual organization, Global/local processing, Individual differences

## Abstract

Individual differences in cognitive processing have been the subject of intensive research. One important type of such individual differences is the tendency for global versus local processing, which was shown to correlate with a wide range of processing differences in fields such as decision making, social judgments and creativity. Yet, whether these global/local processing tendencies are correlated within a subject across different domains is still an open question. To address this question, we develop and test a novel method to quantify global/local processing tendencies, in which we directly set in opposition the local and global information instead of instructing subjects to specifically attend to one processing level. We apply our novel method to two different domains: (1) a numerical cognition task, and (2) a preference task. Using computational modeling, we accounted for classical effects in choice and numerical-cognition. Global/local tendencies in both tasks were quantified using a salience parameter. Critically, the salience parameters extracted from the numerical cognition and preference tasks were highly correlated, providing support for robust perceptual organization tendencies within an individual.

**Supplementary Information:**

The online version contains supplementary material available at 10.3758/s13423-022-02234-6.

## Introduction

A fundamental question in perception is whether humans attend to a visual scene as a whole or whether they focus on its details. A visual object can be viewed as a multilevel hierarchical structure of parts and wholes (Palmer, 1977). Global/local processing reflects the priority of visual processing between these two levels (Navon, 1977; Wagemans et al., 2012a, b). While earlier studies have looked at group effects (e.g., Navon global-precedence theory (Navon, 1977), more recent studies have focused on individual differences in global/local processing tendencies (Chamberlain et al., 2017), which are thought to relate with narrow versus broad attentional focus (Dale & Arnell, 2010, 2013, 2015). Moreover, it has previously been shown that global/local processing style varies as a function of experience (Caparos et al., 2013), culture (Caparos et al., 2012; Davidoff et al., 2008), and skilled behavior (Lewis & Dawkins, 2015), suggesting that there is a large variation across individuals in the tendency for global/local processing. Recently, individual differences in global versus local processing have been suggested to underlie a wide range of differences in cognition, in fields as diverse as decision-making, social-judgments and creativity (Chamberlain et al., 2013; Dijkstra et al., 2013; Drake & Winner, 2011; Förster et al., 2008; Stoesz et al., 2007; Zmigrod et al., 2015). Furthermore, such differences are suggested to account for some of the cognitive pathologies in autism (De Martino et al., 2008; Van Eylen et al., 2018) and schizophrenia (Bellgrove et al., 2003).

Many studies have used the Navon task (Navon, 1977, 1981) to quantify individual differences in global/local processing style (Dijkstra et al., 2013; Förster & Higgins, 2005; Hedge et al., 2018). In this task, subjects are presented with hierarchical letters (e.g., small Ss in the shape of a large H, and the other way around) and are asked to categorize based on either the global or the local shape (and ignore the other shape, which is designated as task irrelevant). In a recent study, Hedge et al. (2018) have examined test–retest reliability in several global–local indices extracted from the Navon task, such as the difference in reaction time (RT) between conditions in which attention was deployed to the local level (RT local) and to the global level (RT global). They reported low test–retest reliability as there was no significant correlation in this global/local index across the two sessions. Interestingly, there was another index that did show high test–retest reliability: the fraction of errors on only the local task. However, such a measure does not contrast local processing with global processing, and is very likely confounded with the ability of the subject to focus attention on the task at hand (local) and filter out the irrelevant dimension (global) (Mevorach et al., 2006). Moreover, Milne and Szczerbinski (2009) showed that there were relatively low inter-subject correlations across 14 different tasks that were aimed to measure global/local processing style. Dale and Arnell (2013) came to a similar conclusion in which test–retest reliability across three different global/local processing tasks was relatively low. Finally, it was shown that global/local processing style as measured using two variations of the Embedded Figures Test (EFT) (Mumma, 1993; Witkin et al., 1971) showed low test–retest reliability with global/local processing style as measured using the Navon task (Chamberlain et al., 2017).

To provide a robust demonstration of individual differences in global/local processing, it is important to first show that the global/local measure shows a high correlation even under different task contexts. Here, we developed a new measure of global/local processing, and we show that it is stable (i.e., shows high inter-subject correlation) across two different task contexts: numerical cognition task and preference task with mixed gambles. Importantly, we use the exact same stimuli in both tasks, because different types of stimuli may induce differences in the global/local processing that could mask what could otherwise be robust individual differences.

In particular, we propose a novel method (based on Navon stimuli) to quantify individual differences in global/local tendencies in a numerical and a preference task. The key difference in our novel method is that we do not set one of the stimuli (global or local level) as *relevant* and the other as *irrelevant*, which is the case in the standard procedure (Navon, 1977, 1981). Rather, we make both stimulus levels task-relevant, by setting them in opposition and asking subjects to carry out a type of comparison between them. Thus, subjects had to perceive and attend to both levels of the hierarchical stimuli at the same time. This enabled us to directly contrast and examine each subject’s tendency to spontaneously focus on either the global or the local features of each stimulus, under the rationale that if a subject has a tendency towards global or local processing, this should be reflected in the relative salience of the global/local information when both are task relevant. To extract for each subject this global–local salience tendency parameter, we carried out computational modeling.

Each subject performed the two tasks in two separate sessions 1 week apart. We then utilized the individual differences in these tasks and correlated, across subjects, between the global/local tendencies estimated from each of the tasks.

## Materials and methods

### Subjects

Thirty subjects were recruited for the current study (mean age = 23.25 years, SD = 4.09; 16 female), in exchange for course credits. Their sight was normal or corrected to normal. All subjects gave written informed consent. All methods were performed in accordance with the relevant guidelines and regulations of the Declaration of Helsinki. All experiments were approved by the committee for the protection of human subjects at Tel Aviv University.

### Stimuli and apparatus

The stimuli were exactly the same in both tasks. Subjects sat in a dark room and the stimuli were presented on a 17-in. monitor (1,280 × 1,024 pixels) with a refresh rate of 75 Hz. The viewing distance was 70 cm, so that each centimeter on the screen represented 0.81° of visual angle. All stimuli appeared against a black background. Each compound figure was spatially arranged on a 5 × 7 grid to form a global number that subtended 3.16° × 6° of visual angle (in width and height, respectively). Each local number subtended 0.35° × 0.56° of visual angle (in width and height, respectively). The inter-element distance was 0.35°. The compound stimuli could appear at one of two possible locations with a distance of 1.50° to the left or right of the fixation mark along the midline. The mask was a 5 × 7 grid of filled white dots, subtended identical size for both local and global elements as the target stimulus. The experiment was run using E-Prime v.2.

## Session 1: Numerical cognition task

### Materials and procedure

**Fig. 1:**
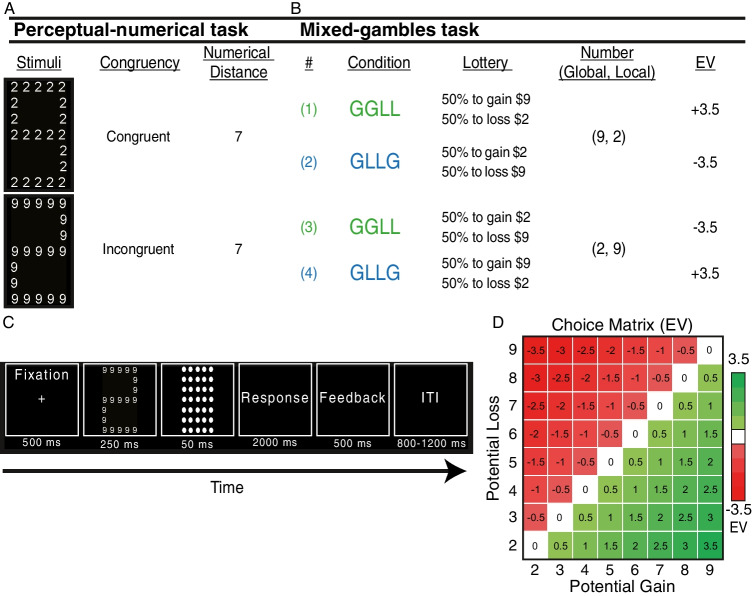
Experimental design. **A** *Stimuli:* An illustration of the compound figure used for both tasks. Features of the Numerical cognition task. Each stimulus was characterized by *Congruency *and by *Numerical Distance*. A stimulus was considered congruent if the global number (big in physical size) also had a larger numerical value, and incongruent if the global number had a lower numerical value. *Numerical Distance *was defined as the numerical difference between the global and local levels (‘7’ in the example stimulus). **B** Features of the Preference task. *Condition:* The task included two conditions: (1) the number in the global level represented the potential gain amount, whereas the number in the local level represented the potential loss amount (GGLL); and (2) vice versa, in which the number in the global level represented the potential loss amount, whereas the number in the local level represented the potential gain amount (GLLG). *Lottery:* A description of the possible gambles based on the condition. *EV:* The expected value of the example lottery in each of the conditions. **C** Single-trial procedure for both tasks. **D** The choice matrix representing all the lotteries and their corresponding EVs that were used in the preference task

We developed a behavioral paradigm based on Navon's figure (Navon, 1977) that included numbers instead of letters (cf. Della Libera & Chelazzi, 2006). The stimuli were composed of a combination of all possible pairs of numbers between 2 and 9 (excluding combinations in which the global and the local values were equal because neither of the levels is larger) for a total of 56 unique combinations. For each compound figure, we computed the numerical distance by calculating the absolute difference between the global and the local numbers (*numerical distance*). In addition, we defined *congruent* (*incongruent*) trials, as those in which the number at the global (local) level was larger than the number at the local (global) level (Fig. 1A).^1^On each trial, subjects were instructed to look at the compound figure and decide whether the number appearing at the global level or at the local level was larger. Figure 1C illustrates the timeline of the experiment. We recorded RTs and accuracy for each response. The task included 448 trials (split into four blocks of 112 trials each). Within each block, the order of stimuli presentation was random. Each unique compound figure was presented eight times in total. After subjects completed the session, we gave them an endowment of ₪40 (equivalent to ~ $12) and asked them to bring this amount of money to the second session.

## Session 2: Preference task

### Materials

We used the exact same compound figure as in session 1. However, as can be seen in Fig. 1B, each compound figure represented a 50–50 chance of winning or losing some amount of money against a fixed value of zero. We used the same number matrix as in the first task but we also included the set of all equal-number trials (e.g., large 5 composed out of small 5 s) for a total of 64 unique compound figures. As shown in Fig. 1D, the risky gamble in each trial had one of eight potential gains ranging from + $2 to + $9, and one of eight potential losses ranging from − $2 to − $9, both sampled in increments of $1. Although the different lotteries were expressed in US dollars, it was clear to our subjects that they were playing for Israeli shekels. That is, we multiplied by 4 all payoffs (dollar-shekel exchange rate) at the time of payment.

For each lottery (compound figure), we computed the expected value (EV) as follows:

$${EV}_{i}=p\times {Amount}_{G\left(i\right)}+\left(1-p\right)\times {Amount}_{L\left(i\right)}$$

where *EV*_*i*_ is the expected value for trial *i*, *Amount*_*G(i)*_ and *Amount*_*L(i)*_ are the monetary amounts that can be gained or lost, respectively, and *p* is the probability of winning (always 0.5 in our experiment).

### Procedure

A week after the numerical cognition task, subjects came to the lab again and conducted the preference task. Subjects were asked to accept or reject a series of mixed gambles with equal probability (50%) of winning or losing a variable amount of money against a fixed amount of zero. Subjects responded with a key-press on the keyboard. The timeline was identical to the timeline of the numerical cognition task (Fig. 1C). However, in the beginning of each block we informed the subject regarding the condition type of that block (Global Gain-Local Loss; GGLL) or vice versa (Global Loss-Local Gain; GLLG), that is, whether the global (local) level represented a gain or a loss. Subjects completed a total of 512 trials, 256 trials per condition. There were four blocks (128 trials per block) with two blocks for each condition. The order of block presentation was counterbalanced across subjects. Within each block, the order of lottery presentation was random. Overall, each unique lottery was presented eight times, four times for each condition. To introduce incentive-compatible payoffs, our subjects were endowed with ₪40 (equivalent to ~ $12) on the first session (at the end of the numerical cognition task), and knew that at the end of the experiment, the computer would randomly pick one trial and they could win, lose, or stay with the endowment they received based on the choice they had made on that trial.

### Computational modelling

1. *Preference task* – The probability to accept/reject the lottery was calculated using the following equations:

1 $$p\left(accept\right)=\frac{1}{1+{e}^{-\left(\beta \cdot U\left(lottery\right)\right)}}$$

2 $$p\left(reject\right)=1-p\left(accept\right)$$

where $\beta$ is a free parameter indicating sensitivity to utility (i.e., inverse of noise), and the utility of each lottery was defined as:

3 $$U\left(lottery\right)=\left\{\begin{array}{c}Amoun{t}_{G}\cdot {\theta }_{preference}-Amoun{t}_{L}\cdot \lambda ,GGLLcondition\\ Amoun{t}_{G}-Amoun{t}_{L}\cdot \lambda \cdot {\theta }_{preference},GLLGcondition\end{array}\right.$$

where $Amoun{t}_{G}$ and $Amoun{t}_{L}$ represent the gain and loss amounts, respectively, $\lambda$ is a loss aversion parameter (Tversky & Kahneman, 1992), and ${\theta }_{preference}$ is a free parameter representing the value modulation resulting from global/local biases, such that ${\theta }_{preference}>1$ indicates a global-bias, ${\theta }_{preference}<1$, indicates a local-bias, and ${\theta }_{preference}=1$, indicates a lack of any global/local bias. Thus, if a subject has a global bias, values presented as global numbers (either gains or losses) are increased by a factor of $\theta$, and values presented as local numbers are reduced by the same factor. If a subject has no global/local bias, the factor is equal to 1 (and thus the value function is similar to the standard Prospect Theory function with $\alpha$ = 1; Kahneman & Tversky, 1979). Model parameters were fitted individually for each subject.

**Fig. 2:**
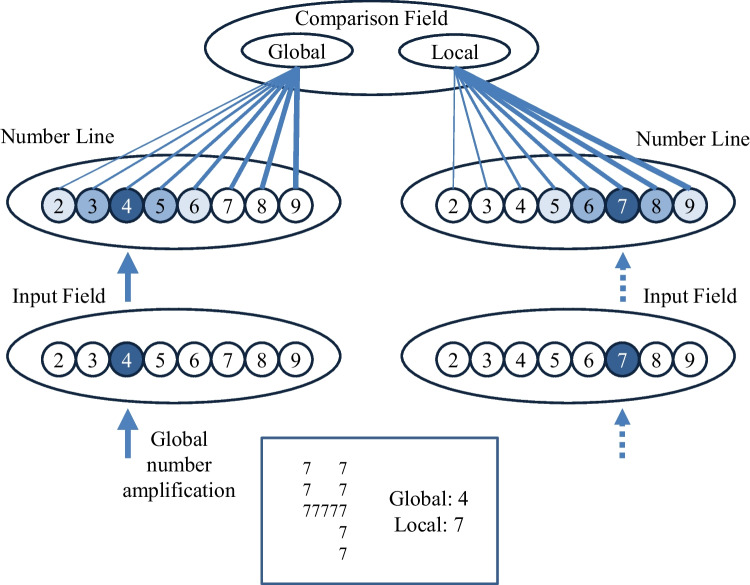
Computational model for the *Numerical cognition task*. The first (bottom) layer is the input layer, which corresponds to Arabic numbers based on a place-coding system. The arrows represent the strength of the input. The solid line corresponds to the input of the global number, which is amplified relative to the local number (dashed arrow), assuming that θ_perceptual_ is higher than 1 (see Eq. 7). The second (middle) layer is a hidden layer, which activates the corresponding unit with maximal strength, and neighboring units with gradually decreasing strength as a function of distance. The third (top) layer is the response layer according to the global/local information. Blue lines correspond to the connections between the second to third layers. Line thickness is proportional to connection strength. Adapted based on Verguts et al. (2005)

2. *Numerical cognition task* – The performance in the numerical cognition task was modeled using a variant of the neural network model developed by Verguts et al. (2005). The model has three layers (see Fig. 2 for an illustration):

i) Input field layer – This layer consists of a set of units corresponding to Arabic numbers based on place-coding system (the number ‘1’ activates the first unit, the number ‘2’ activates the second unit, and so on; see Fig. 2). The activation in the input field was implemented as follows:

 

4 $$d{X}_{i}\left(t\right)/dt= -Xi(t)+Ii(t)$$

where $i$ is the index of each unit ($1\le i\le 15$, as in Verguts et al., 2005) and $t$ is the time-step. ${X}_{i}(t)$ is the activation of the of the $i$-th unit at time t (e.g., ${X}_{4}$ is the activation of fourth unit in the input field, which is activated by the number ‘4’). $Ii(t)$ is an indicator function that is equal to 1 in case the number $i$ is presented and to 0 otherwise (e.g., ${I}_{4}(t)$ is equal to 1 if the number ‘4’ is presented and to 0 otherwise). Note that $-{X}_{i}(t)$ is a decay term, indicating that if the number $i$ is not presented (i.e., $Ii(t)$ = 0) the activation of ${X}_{i}(t)$ exponentially decreases to zero. If the number *i* is presented ($Ii(t)$ = 1), then the activation ${X}_{i}(t)$ increases toward an asymptotic value of 1. We used separate input fields for the global and local numbers.

In order to model the global/local tendencies of the participants, we multiplied the indicator function ($Ii(t)$) of the global number input field by a free parameter labeled ${\theta }_{perceptual}$, so that:

5 $$d{X}_{i}\left(t\right)/dt= -Xi\left(t\right)+{\theta }_{perceptual}\cdot Ii(t)$$

Similar to the preference-task model, if the value of ${\theta }_{perceptual}$ is higher than 1, then the activation of the global number is increased, indicating a global bias. In contrast, if the value of ${\theta }_{perceptual}$ is lower than 1, then the activation of the global number is decreased, indicating a local bias. Finally, a ${\theta }_{perceptual}$ value of 1 indicates a lack of bias.

ii)  Hidden number line layer – This layer also consists of units corresponding to Arabic numbers based on a place-coding system. It is fed with the unit activation from the first layer, and activates the corresponding unit with maximal strength (e.g., ‘4’ in the global network and ‘7’ in the local network in Fig. 2). In addition, neighboring units (e.g., 2, 3, 5, and 6 if the number ‘4’ is presented) are activated with gradually decreasing strength as a function of distance (that is, the activation of 5 would be higher than the activation of 6). The activation in the number line layer was implemented as follows:

6 $$d{Y}_{j}\left(t\right)/dt= - {Y}_{j}\left(t\right)+{\sum }_{i=1}^{n}{e}^{-\left|i-j\right|}\cdot {X}_{i}(t)$$

where $j$ is the index of each unit ($1\le j\le 15$) and $t$ is the time-step. ${Y}_{j}\left(t\right)$ is the activation of the $j$-th unit at time t. As in the input layer, $-{Y}_{j}\left(t\right)$ is a decay term. $Xi(t)$ has strong influence on ${Y}_{j}\left(t\right)$, when $i = j$ (in this case, ${e}^{-\left|i-j\right|}=1$), but weaker influence the further $i$ and $j$ are from each other (e.g., if $i =4$ and $j=7$ then ${e}^{-\left|7-4\right|}=0.05$). Separate number line fields were used for the global and local numbers.

iii) Response layer – This layer consists of two units corresponding to the global or the local responses. The input to the global (local) response unit was the dot product of the number line activations and their respective weights. To account for the error data in the response layer, we extended the original model by implementing a Diffusion process of evidence integration to a decision boundary (Glickman et al., 2022; Glickman & Usher, 2019; Ratcliff, 2002; Ratcliff & McKoon, 2008; Ratcliff & Rouder, 1998), so that the global and local units accumulated the inputs from their corresponding number line layers across time with the addition of random Gaussian noise, according to the formula:

 

7 $$d{Z}_{k}\left(t\right)/dt= - {Z}_{k}\left(t\right)+ {\sum }_{j=1}^{n}{w}_{j}\cdot {Y}_{j}\left(t\right)+{\varepsilon }_{i}(t), {\varepsilon }_{i}(t)\sim N(0, {\sigma }^{2})$$

where ${Z}_{k}$ is the activation of the global or local units and ${Y}_{j}\left(t\right)$ is the activation of the of the $j$-th unit at time t.${w}_{i}$ was defined using the formula: $1.1-{10}^{j/(n-1)}$, resulting in a concave weighting function similar to the one used by Verguts et al. (2005), ranging between 0.1 to 1 $.$ ${\varepsilon }_{i} is$ a random Gaussian noise. Response is initiated once the difference between the activations of the global and local (${Z}_{1}$ and${Z}_{2}$) units crosses a predefined threshold. At the beginning of each trial, ${Z}_{1}$ and ${Z}_{2}$ were set to 0. Model parameters were fitted individually for each subject.

## Results

### Numerical cognition task

**Fig. 3:**
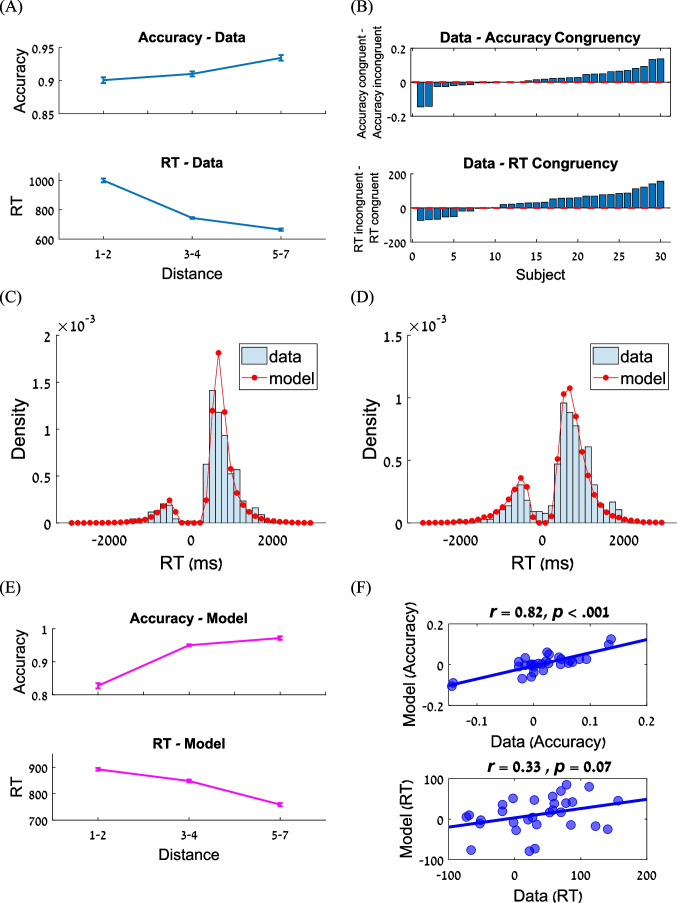
Numerical cognition task. **A** Distance effects in the numerical cognition task for accuracy and reaction time (RT). Error bars correspond to within-subject standard errors of the mean. **B** Individual differences in congruency effects in the numerical cognition task for accuracy and RT. Subjects in the two panels are sorted independently. **C** and **D** RT distributions of two representative participants. The x-axis indicates RTs, and the y-axis indicates the density. Error responses are mirrored on the negative x-axis. Red lines represent simulated RT distributions for each participant, based on 1,000 simulations of each trial. **E** Model-predicted distance effects in the numerical cognition task for accuracy and RT. Error bars correspond to within-subject standard errors of the mean. **F** Correlations between the empirical (a-axis) and predicated (y-axis) congruency effects for accuracy (**top panel**) and RT (**bottom panel**). Each point corresponds to a single participant

In the numerical cognition task, we found the standard distance effects: RTs were faster (*F*(2,58) = 101.72, *p* < 0.001) and accuracy was higher (*F*(2,58) = 11.94, *p* < 0.001) when the two digits (at the global and local levels) were more distant from each other (e.g., 2–9 compared with 4–5; Fig. 3A). Furthermore, most subjects showed a congruency effect (global precedence effect): more correct responses and faster RTs when the global digit is larger in its numerical value than the local digit, compared to the opposite. This effect, however, was subject to considerable individual differences as shown in Fig. 3B. Next, we fitted the computational model of numerical cognition (Verguts et al., 2005) to the data (see Table S1 in the Online Supplementary Material (OSM) for the best fitted parameters of each participant). As can be seen, the model captured the RT distributions well (see Fig. 5C and D for two representative participants and Fig. S1 (OSM) for all participants), and qualitatively accounted for the distance (Fig. 3E) and congruency effects (Fig. 3F) for accuracy and response times.

### Preference task

**Fig. 4:**
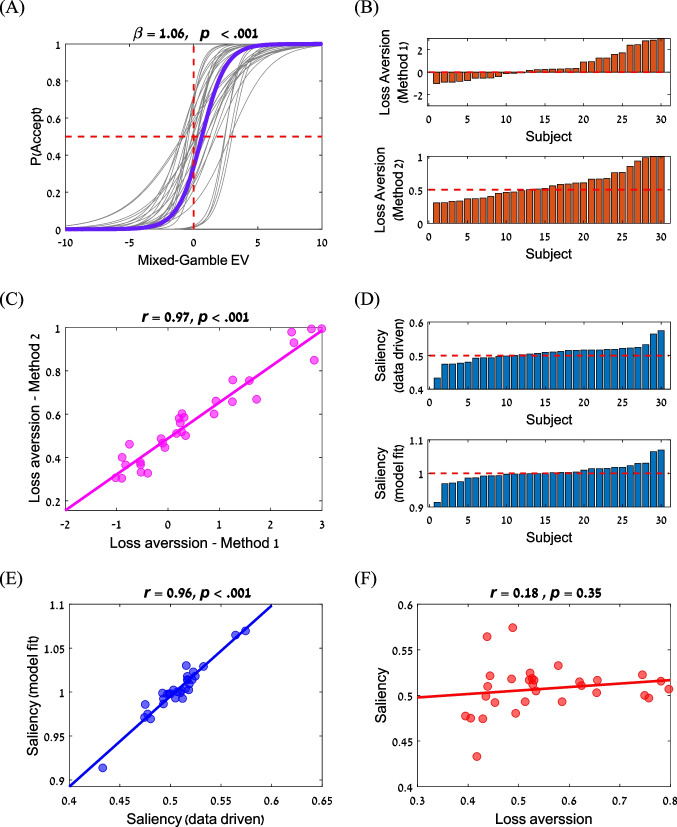
Preference task. **A** Participants were sensitive to changes in EV and demonstrate loss-aversion at the group-level (the EV (expected value of the example lottery in each of the conditions) at indifference point is higher than 0). The solid purple line corresponds to the group fit; grey lines correspond to the fit of individual participants. **B** Individual differences in loss-aversion, estimated as rejection rate for EV = 0 (Method 1; upper panel), and as the acceptance rate for the gamble (Method 2; lower panel); Red dashed lines indicate loss neutrality. Subjects in the two panels are sorted independently. **C** Correlation between loss-aversion as estimated using both methods. **D** Individual differences in data-driven global–local bias (upper panel), and global–local bias based on a computational method model fit (lower panel). Red dashed lines indicate lack of bias. **E** Correlation between global–local bias as estimated using both methods (data driven and model fit). **F** No correlation was found between the global–local bias and loss aversion

In the preference task, participants showed sensitivity to the EV of the mixed gambles. A mixed-effect logistic regression on the choice data, with the EV of the mixed-gamble as a predictor and with random intercepts and slopes at the participant level, showed that the preference for the mixed gamble over the zero-default option increased with the EV of the lottery (*β* = 1.06, *p* < 0.001; Fig. 4A). In addition, participants show a typical loss-aversion behavior, which was measured using two methods: (i) the EV value at the indifference point (method 1, Fig. 4B/top), *t* = 2.42, *p* = 0.021, and (ii) the overall acceptance rate for the gamble option (method 2, Fig. 4B/bottom), *t* = 2.34, *p* = 0.026. Both measures show a high correlation between them (*r* = 0.97, *p* < 0.001), but were subject to high individual differences (Fig. 4C).

Our central focus is to examine individual differences in the level of their global/local bias (i.e., their tendency to demonstrate a global precedence effect). We extracted these biases in two ways: (i) a simple data-driven method, and (ii) a computational method. The data-driven method extracts for each subject the fraction of trials, in which s/he responded only on the basis of the global level, i.e., the fraction of trials in which the subject accepted the gamble when the global number represented a gain, and rejected it when the global number represented a loss independently of the actual magnitude of the gain or loss (e.g., accept the lottery 6/global and reject the lottery -7/global and 6/local). The computational method fits to each subject a salience to global (bias) parameter, $\theta$ which is larger than 1 for an individual with a global bias and smaller than 1 for an individual with a local bias (see *Computational models*). While on average at the group level there was only a small global/local bias (${\theta }_{median}=1.001$), we found large individual differences (Fig. 4D; see Table S2 (OSM) for the best fitted parameters of each participant), which were highly correlated between the data-driven and computational methods (Fig. 4E). Importantly, no correlation was observed between the individual tendency for loss aversion and for global/local bias (*r* = 0.18, *p* = 0.35; Fig. 4F), which suggests that these biases stem from different underlying cognitive mechanisms.

### Across-domain global–local individual differences

**Fig. 5:**
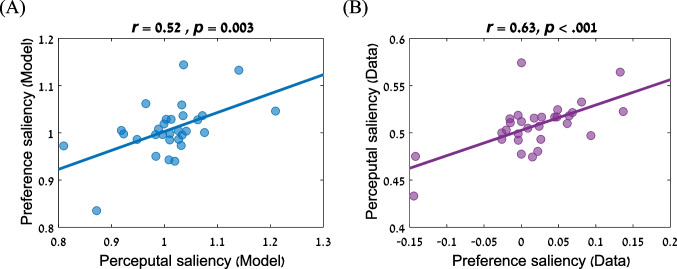
Correlations of global/local processing style between the perceptual and preferential tasks. **A** Model driven approach: The perceptual saliency parameter (${\uptheta }_{\mathrm{perceptual}}$) is associated with the preference saliency parameter (${\uptheta }_{\mathrm{preference}}$). **B** Data-driven approach: The behavioral preference saliency measure was associated with the perceptual one

The central aim of our study was to test for correlations between measures of global/local processing across the perceptual and preference domains. As shown in Fig. 5, there is a robust correlation between the individual differences in the two domains. This is shown both via the computational-based estimated saliency parameters (Fig. 5A), and by using the data-driven approach, in which we correlated the salience effect in preference to the salience effect in perceptual choice (quantified as accuracy-congruency in the numerical cognition task; Fig. 5B).

A possible alternative explanation for the correlations found between the global/local measures in both domains is that the subjects deployed a similar strategy in the numerical cognition and preference tasks. For example, in the preference task the subjects could potentially use a simple heuristic of accepting a gamble if the potential gain is larger than the potential loss and rejecting it otherwise. This heuristic exactly maps to the process required in the numerical cognition task. To rule out this common strategy explanation, we demonstrate a qualitative difference in how the subjects performed the two tasks, indicating different underlying mechanisms. In particular, we show that the subjects showed loss aversion in the preference task but not in the numerical cognition task (see Fig. S2 (OSM)). Following this analysis, we also compared the quantitative fit of a model based on the alternative heuristic to the preference model used in this study (see Computational modelling/Preference task Eqs. 1–3). The results indicate that the preference model that we used shows a much better fit for the data then the heuristic-based model (AIC_model_ = 9,856 and AIC_heuristic_ = 12,024, lower values indicate a better fit; see Supplementary Results (OSM) for further details). These results thus provide both qualitative and quantitative support for differences in the performance of the numerical cognition and preference tasks.

## Discussion

In this study we modified the classic Navon task so that subjects freely focus on both of the processing levels while they make a magnitude comparison between the levels (in the numerical cognition task) or while they combine the information across levels (representing gain/loss of a mixed gamble lottery) to form a value estimate (in the preference task). We designed this novel task to measure the participant's own (spontaneous) processing style that is not masked by attentional control differences (responding to the task-relevant dimension and ignoring the other one).

We showed that in both tasks there is a high variability in global/local bias across subjects, which is in line with previous studies (Dale & Arnell, 2010, 2013, 2015; De-Wit & Wagemans, 2013). Most subjects showed a global bias, that is, preference for information nested at the global level, whereas some subjects had no bias at all, and some demonstrated a local bias, that is, an enhanced ability for processing low-level information. This high variability across subjects probably contributed to our small group average effects. We found a small average global bias in which subjects tend to accept the lottery when the global level is a gain compared to when the global level is a loss. Importantly, the global bias was evident even after controlling for the effect of EV on choice, demonstrating an effect of perceptual processing on value-based choice (Anderson, 2013; Glickman et al., 2019; Hickey et al., 2010; Milosavljevic et al., 2012; Towal et al., 2013).

These findings emphasize the importance of not only relying on group averages but rather examining heterogeneity across subjects. Approximately a third of the subjects demonstrate a local bias, which is in contrast to our finding for the group and is opposite to the common finding reported in the literature (Flevaris & Robertson, 2016; Kimchi, 1992; Navon, 1977; Wagemans et al., 2012b).

In the numerical cognition task, we found that subjects were also affected by the numerical distance of the numbers, as has been shown in previous studies (Dehaene et al., 1990; Moyer & Landauer, 1967). They were faster and more accurate as the distance between the numbers increased. Moreover, on average, subjects showed a small tendency for global precedence (which in our task was the same as a congruency effect) in which they tend to be more accurate and faster when the global digit was larger than the local digit, compared to the opposite. This is in line with previous studies that demonstrated a numerical congruency effect (Dehaene, 2011; Dehaene et al., 1990; Henik & Tzelgov, 1982; Moyer & Landauer, 1967), but we substituted the dimension of size with global/local presentation. Across subjects there was no correlation between loss-aversion and saliency parameters, suggesting that these effects probably result from different underlying mechanisms. However, although previous studies demonstrated the positive effect of saliency in increasing the propensity to choose a more salient option with equal value (Milosavljevic et al., 2012; Towal et al., 2013), and that previously rewarded locations are attended to faster and more accurately (Anderson, 2013; Hickey et al., 2010), our findings suggest that on a subject level, loss aversion is not directly influenced by our saliency manipulation and that global/local processing tendency is not directly linked to the mechanisms inducing loss aversion.

Our central result showed that there is a high correlation between the local/global measures in the two tasks. The high correlation across tasks indicates that global/local processing style within an individual is robust when the goal of the task changes. We suggest that the robust correlation we found was due to our novel design in which subjects were free to focus on either of the processing levels, revealing their “true” subjective trait regarding their processing style, irrespective of the specific task. Note that our results need to be qualified for the specific order of tasks that we used here (the numerical cognition task was performed first). The reason we used this specific order was twofold: First, we used real money in the preference task and we did not want the earnings of the participants to affect their behavior in the numerical task. There was a lower risk that performance in the numerical cognition task would affect behavior in the preference task, since participants did not get any feedback in this task. Second, at the end of the numerical session subjects received money for playing in the preference task a week later. We did that because they could lose money and wanted them to feel as if it is their own money. Thus, we could not switch the order of the tasks.

The robust correlations hold whether we use simple behavioral measures or whether we use the parameters extracted from our computational modelling (Fig. 5A and B). Using the latter framework allowed us to decompose task-performance measures (accuracy and RTs) into interpretable psychological processes, to isolate the effects of saliency in the numerical cognition and preference tasks, and to examine the association between them. In particular, we modeled the preference task using a logit model, in which the amounts were modulated by global precedence. The numerical cognition task was modeled using a biologically plausible neural network based on a place-coding system (Verguts et al., 2005), and in which the activations in the input field are modulated by global precedence.

To conclude, we demonstrated a robust measure of global/local individual differences, which we suggest to be crucial in order to examine complex cognitive mechanisms and their influence across domains. Future studies should examine if the individual differences in global/local processing that we have proposed predict differences in the performance of a wide range of other cognitive domains such as creativity, social judgment, and memory, as well mediating a number of cognitive/clinical pathologies like autism and schizophrenia.

## Supplementary Information

Below is the link to the electronic supplementary material.

Supplementary file1 (PDF 732 KB)

## Data Availability

All data and materials supporting these experiments are available upon request. None of the experiments was preregistered.

## Footnotes

1 We used the terms congruent/incongruent, because the global number (big in physical size) is also larger in numerical value and the local number (small in physical size) is also smaller in numerical value (and vice versa for incongruent trials; see Henik & Tzelgov, 1982).
