# Supplementary material for: The effect of perceptual organization on numerical and preference-based decisions shows inter-subject correlation
Source: Psychon Bull Rev. 2023 Jan 10;30(4):1410–21. doi: 10.3758/s13423-022-02234-6 (PMC10482786; doi:10.3758/s13423-022-02234-6)
Supplement: Supplementary file 1 — Supplementary file1 (PDF 732 KB) [file 13423_2022_2234_MOESM1_ESM.pdf]

Supplementary Information

Numerical Cognition Task – Best Fitted parameters

Table S1. *The best fitted parameters of the numerical cognition task. Values in parentheses correspond to the standard deviation of the best fitted parameters across participants.*

Subject	*Noise*	*Boundary*	*Saliency*
1	0.13	0.13	0.98
2	0.06	0.06	1.01
3	0.27	0.63	1.00
4	0.08	0.08	1.03
5	0.34	0.80	1.01
6	0.21	0.18	0.99
7	0.44	1.00	0.92
8	0.32	0.30	1.06
9	0.33	0.78	0.92
10	0.15	0.15	1.07
11	0.33	0.81	0.96
12	0.34	0.44	1.14
13	0.03	0.04	1.00
14	0.13	0.12	1.04
15	0.11	0.11	1.02
16	0.11	0.15	0.98
17	0.13	0.13	0.87
18	0.38	0.98	1.08
19	0.04	0.05	1.03
20	0.22	0.61	1.03
21	0.30	0.32	0.95
22	0.05	0.06	1.01
23	0.34	0.80	1.01
24	0.33	0.21	0.81
25	0.03	0.05	1.04
26	0.06	0.06	1.03
27	0.11	0.11	1.03
28	0.31	0.75	1.04
29	0.04	0.05	1.00
30	0.31	0.31	1.21
Group-Level	0.20	0.34	1.01
	(0.13)	(0.32)	(0.07)

Numerical Cognition Task – Response time distributions

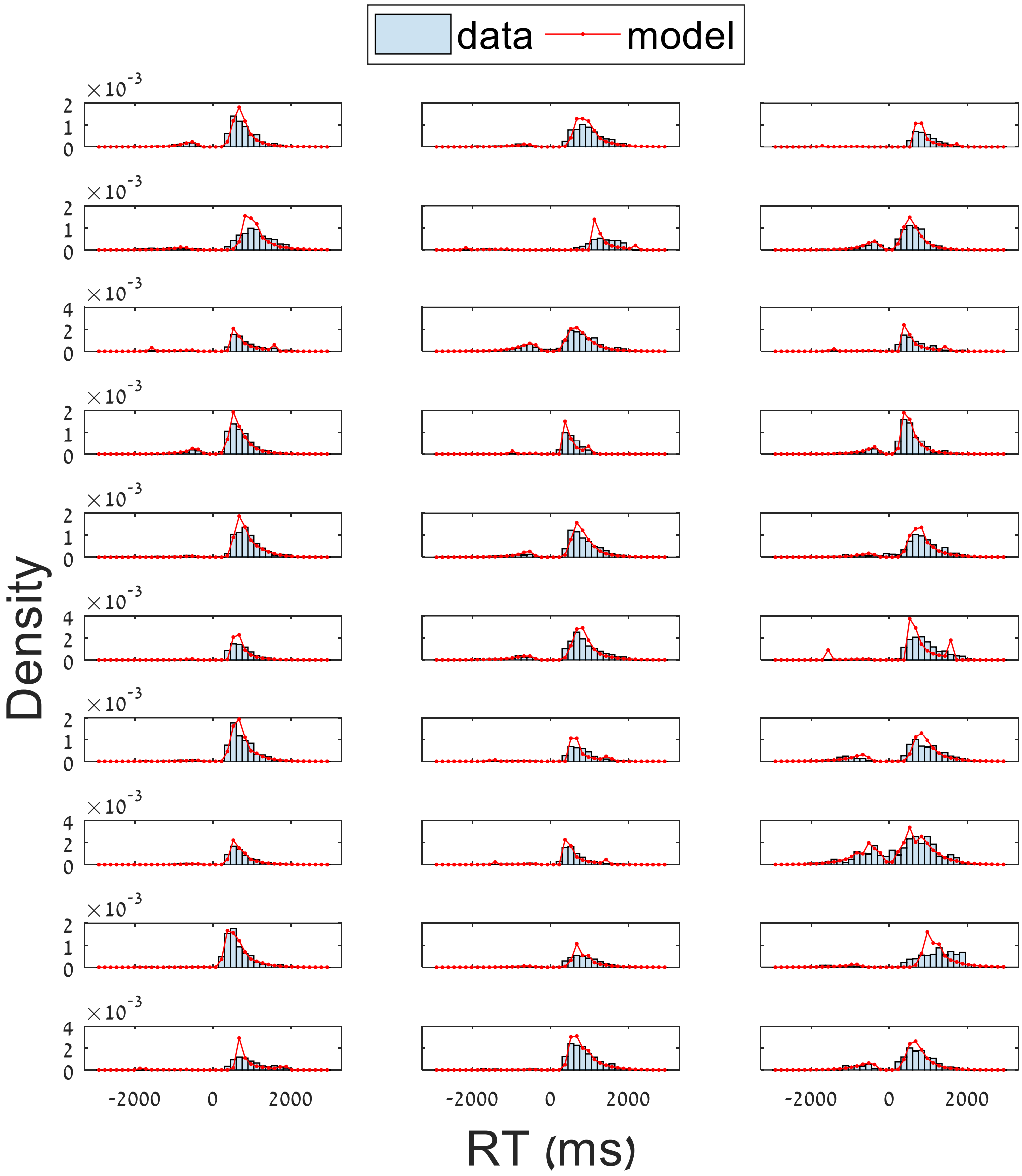

Fig. S1. *Response-Time distributions of all participants in the numerical cognition task. The x-axis indicates Reaction-Times, and the y-axis indicates the density. Error responses are mirrored on the negative x-axis. Red lines represent simulated RT-distributions for each participant, based on 1,000 simulations of each trial.*

Preference Task – Best Fitted parameters

Table S2. *The best fitted parameters of the preference task. Values in parentheses correspond to the standard deviation of the best fitted parameters across participants.*

Subject	*Noise*	*Loss-Aversion*	*Saliency*
1	1.15	0.82	0.95
2	0.49	0.87	0.94
3	3.87	0.93	1.00
4	1.19	0.97	1.00
5	1.85	0.93	1.00
6	0.77	1.20	1.01
7	0.76	1.33	1.01
8	1.57	1.03	1.03
9	2.92	0.96	1.00
10	0.61	1.33	1.04
11	1.29	1.71	1.06
12	0.45	0.87	1.13
13	0.48	1.00	1.03
14	1.03	1.02	1.04
15	1.09	0.99	0.94
16	2.85	1.59	1.00
17	0.98	0.88	0.84
18	2.37	1.81	1.00
19	3.16	1.05	0.99
20	2.09	1.57	1.00
21	0.62	1.00	0.99
22	0.54	1.23	0.98
23	1.05	0.95	1.03
24	1.41	0.88	0.97
25	1.27	0.94	1.14
26	0.80	1.30	0.97
27	0.54	1.12	1.06
28	0.71	1.16	1.00
29	1.89	1.06	1.02
30	0.41	1.60	1.05
Group-Level	1.34	1.13	1.01
	(0.94)	(0.27)	(0.06)

Distinction between the numerical cognition task and the preference task

In this section, we demonstrate a qualitative difference between the numerical cognition task and the preference task, indicating a difference in their underlying mechanisms. In particular, we show that the participants showed loss aversion in the preference task but not in the numerical cognition task. To this end, we use both model-free and model-based analyses.

In the mode-free analysis, we examined the response patterns in trials in which the difference between the absolute value of the global and local numbers was 1. For example, 5 vs. 6 in the numerical cognition task, and 5 (loss) vs. 6 (gain) in the preference task (in the preference task we only analyzed trials in which the smaller number corresponded to loss, so there is a conflict between the positive expected value of the gambles and the possibility of loss). If the judgments of the participants were based on similar mechanisms on both tasks, then choice patterns should be similar as well (i.e., the proportion of choosing the higher number in the numerical cognition task should be similar to the proportion of accepting the gamble in the preference task). However, if participants weight losses higher than gains (i.e., show loss aversion), then the proportion of accepting the gamble in the preference task should be lower than the proportion of correct responses in the numerical cognition task. As shown in the Fig. S2, the proportion of choosing the higher number in the numerical cognition task (M = 0.89) was significantly higher than the proportion of accepting the gamble in the preference task (M = 0.61; $t(29) = 4.49$; $p < .001$), indicating a different underlying mechanism.

In the model-based analysis, we compared the preference task model which is described in the main text (Computational Modelling/Preference task Eq. 1-3), to a model which does not assume loss aversion. Note that the second model was similar to the first one, expect for the λ parameter (which accounts for loss aversion) was set to 1. Note also that the latter model corresponds to a heuristic of deciding whether to accept or reject the gamble based on a comparison between the absolute value of the gain and loss in each gamble (i.e., accept the gamble if the gain is higher than the loss and reject otherwise). The model which assume loss aversion decisively outperformed the which model does not ($AIC_{model\ 1}$ = 9,856 vs. $AIC_{model\ 2}$ = 12,024, AIC difference higher than 10 is considered a strong evidence in favor of the model with the lower AIC value), suggesting a different cognitive processing of the stimuli in both tasks.

The model-free and model based analyses suggest that in the preference task, but not in the numerical cognition task, the value of the alternatives was subject to loss-aversion – a process that is characteristic to subjective-utility of mixed gambles. These results rule out the possibility that the subjects used the same strategy in both tasks.

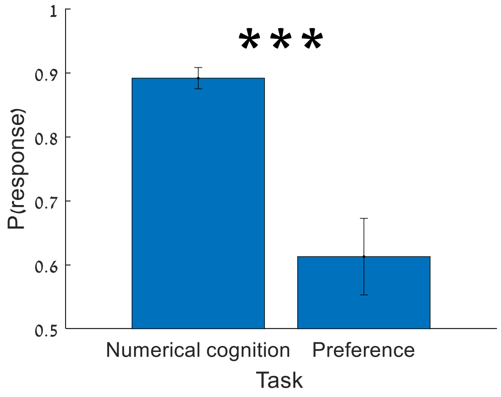

Fig. S2. *The Probability of an accurate response in the numerical cognition task and of an accept response in the preference task. *** $p < .001$.*
